# Diabetes and Cardiovascular Complications: The Epidemics Continue

**DOI:** 10.1007/s11886-021-01504-4

**Published:** 2021-06-03

**Authors:** Raquel López-Díez, Lander Egaña-Gorroño, Laura Senatus, Alexander Shekhtman, Ravichandran Ramasamy, Ann Marie Schmidt

**Affiliations:** 1 Diabetes Research Program, Department of Medicine, New York University Grossman School of Medicine, 435 East 30th Street, Science Building, Room 615, New York, NY 10016 USA; 2 Department of Chemistry, The State University of New York at Albany, Albany, NY USA

**Keywords:** Diabetes, Cardiovascular disease, Epigenetics, Interferon pathways, Mitochondrial dysfunction, Therapeutic agents

## Abstract

**Purpose of Review:**

The cardiovascular complications of type 1 and 2 diabetes are major causes of morbidity and mortality. Extensive efforts have been made to maximize glycemic control; this strategy reduces certain manifestations of cardiovascular complications. There are drawbacks, however, as intensive glycemic control does not impart perennial protective benefits, and these efforts are not without potential adverse sequelae, such as hypoglycemic events.

**Recent Findings:**

Here, the authors have focused on updates into key areas under study for mechanisms driving these cardiovascular disorders in diabetes, including roles for epigenetics and gene expression, interferon networks, and mitochondrial dysfunction. Updates on the cardioprotective roles of the new classes of hyperglycemia-targeting therapies, the sodium glucose transport protein 2 inhibitors and the agonists of the glucagon-like peptide 1 receptor system, are reviewed.

**Summary:**

In summary, insights from ongoing research and the cardioprotective benefits of the newer type 2 diabetes therapies are providing novel areas for therapeutic opportunities in diabetes and CVD.

## Introduction

Despite sustained public health messages targeting modifiable risk factors, such as attention to body weight, dietary and nutritional factors, cessation of smoking, and enhanced physical activity, the incidence of diabetes continues to rise. The International Diabetes Federation (IDF) has estimated that worldwide, approximately 463 million persons have diabetes, and of these, 91% suffer from type 2 diabetes (T2D) [1]. Furthermore, the IDF predicts that the number of cases of persons with diabetes, worldwide, will exceed 700 million by 2045 [1], thereby underscoring the importance of “flattening the curve” in this epidemic.

Although it might well be presumed that such rises are accounted for by the absolute rise in population, this appears not to be the case. For example, during the period from the 1970s to the first decade of the 2000s, the incidence of diabetes per 1000 persons increased from 3.0 to about 5.5 based on data reported by the Framingham Heart Study (FHS) [2]. Furthermore, that report also indicated that the incidence of diabetes over that time frame was higher in male vs. female persons, by a factor of about 1.61 [2]. T2D imparts many adverse consequences to the affected persons; overall, it is estimated that having T2D reduces life expectancy by up to 10 years, and the main cause of mortality in the affected persons is death due to cardiovascular disease (CVD) [3]. Recently, in the context of the current pandemic triggered by SARS-CoV-2 infection (COVID-19), it has been reported that persons with diabetes and infected with this virus have a worse prognosis and experience greater severity of disease [4••, 5••, 6••, 7••, 8••].

Because hyperglycemia is the defining feature of diabetes, much attention has been paid to determining if strict glycemic control affords long-term benefit against the cardiovascular complications of this metabolic disorder.

### Type 2 Diabetes (T2D), Strict Glycemic Control, and Cardiovascular Disease

In T2D, the landmark United Kingdom Prospective Diabetes Study (UKPDS) tested the effects of intensive vs. conventional diabetes therapies with respect to three aggregate endpoints: (1) any diabetes-related endpoint, (2) diabetes-related death, and (3) all-cause mortality and reported that strict control of hyperglycemia in T2D was not associated with a significant decrease in the risk of macrovascular complications, but that most of the risk reduction for any of the diabetes-related aggregate endpoints was due to a 25% risk reduction in microvascular endpoints (including the need for laser photocoagulation) [9]. In work reviewing the body of literature on this question, it was concluded that the effects of strict glycemic control were more relevant for certain and limited microvascular outcomes not necessarily CVD [10]. In the case of CVD, the following conclusions appear to be the case: first, strict control of hyperglycemia in T2D reduces the risk of nonfatal myocardial infarction by about 15% but exerts no overall benefit on all-cause or cardiovascular-related mortality [10]. Indeed, the results of the landmark ACCORD trial cemented the sentiment that strict control of hyperglycemia might, in fact, impart adverse consequences; in ACCORD, the subjects randomized to strict vs. conventional control of glucose levels experienced increased total and CVD-related mortality (along with higher weight gain and hypoglycemia) [11]. When the evidence for benefits, or not, of strict glycemic controls was systematically gathered and analyzed, it was concluded that prior to the ACCORD trial, 47–83% of published studies endorsed this therapeutic strategy; after ACCORD, however, a fewer number of reports (21–36%) advocated for benefits of rigorous glycemic control [11].

### Type 1 Diabetes (T1D), Strict Glycemic Control, and Cardiovascular Disease

In T1D, despite the advances in glycemic control and approaches to the management of CVD, persons with T1D continue to be at higher risk for CVD when compared to age-matched control persons without diabetes [12, 13]. The landmark Diabetes Control and Complications Trial (DCCT)/Epidemiology of Diabetes Interventions and Complications (EDIC) studies (original subject number, 1441) concluded that with respect to macrovascular complications of diabetes, a mean of approximately 6.5 years of intensive vs. standard glycemic control was able to significantly reduce the risk of the macrovascular complications of diabetes [14]. Although hypoglycemic episodes were a side effect, it was determined that these adverse effects did not impart long-term consequences, such as on cognition [15].

When the DCCT/EDIC subject cohort was analyzed over a 29 year period, it was observed that there were 120 cases of major adverse cardiovascular events (MACE) and 239 cases of any CVD-related outcome [16]. Over time, other factors moderated the effects of glycemia, such as higher pulse, higher triglycerides, use of calcium channel blockers, and presence of neuropathy enhanced the effect of glucose levels on any CVD, but with respect to MACE, the effect of glucose was moderated by higher pulse and triglyceride levels, albumin excretion rate, hypertension, and no known family history of T2D [16]. The authors concluded that attention to these other factors, most notably those that are modifiable, might reduce the impact of glycemia on the risk for CVD in T1D.

What was notable about the results of the DCCT/EDIC study was that the benefits of the strict control of hyperglycemia, particularly on microvascular complications, lasted about 12–15 years beyond the time period during which the hemoglobin A1c (HbA1c) levels between the two groups of subjects (intensive vs. conventional control) eventually became indistinguishable, suggesting that the period of elevated levels of blood glucose in the conventional treatment group imparted an adverse “metabolic memory” [17, 18]. Recently, Lachin, Nathan, and colleagues suggested that after this period of 12–15 years, the benefits of the strict glycemic control on metabolic memory appear to wane, and after that time, a period of “metabolic amnesia” ensues [19].

Collectively, for T2D and T1D, on account of the possible harmfulness and limited long-term benefits of strict control of hyperglycemia, these considerations underscore the importance of continuing efforts to identify new therapeutic opportunities for the prevention and management of CVD in disorders of hyperglycemia. In the sections to follow, this Review will chronicle selected updates in representative areas of investigation into the mechanisms of accelerated CVD in diabetes. Specifically, recent updates in the field of epigenetics have provided direct evidence for links between DNA-methylation (DNA-me) and diabetic complications. Roles for high glucose and its immediate downstream sequelae in the upregulation of interferon signaling pathways are considered, and finally, updates into the mechanisms by which high glucose causes mitochondrial dysfunction, thereby affecting multiple cell types affected in CVD, such as cardiomyocytes, vascular cells, and immune cells, are presented. In terms of recent therapeutic advances, the roles for sodium glucose cotransporter-2 (SGLT2) inhibitors and agonists of the glucagon-like peptide 1 receptor (GLP1R) in cardioprotection are discussed.

## Epigenetics: Long-Lasting Memory and Consequences to the Cardiovascular System

Modulation of the epigenome has been studied in the context of diabetic complications; spurred by the findings of the DCCT/EDIC and the concept of metabolic memory, this field in the area of diabetes arose to identify mechanisms for the long-lasting effects of elevated levels of blood glucose on macro- and microvascular complications of the disease. Examples of specific areas for the study of the human epigenome include the following: first, DNA-me has been studied in the context of high glucose and diabetes; DNA-me is usually associated with gene repression. Second, post-translational modifications (PTMs) of histone tails in chromatin (methylation and acetylation) have been probed in the context of high glucose and diabetes; these PTMs may lead to either gene activation or repression [20–24]. These epigenetic changes have been studied in multiple cell types that are highly germane to diabetic cardiovascular, as well as other complications; these include vascular cells and myeloid/whole blood cells.

### Vascular Cells

Previous work identified mechanisms by which cultured vascular cells underwent sustained modulation of gene transcriptional profiles after transient exposure to high-glucose conditions. Some of the key discoveries in this area identified new roles for methyltransferases in the epigenetic processes.

Transient exposure to hyperglycemic conditions in cultured cells imparts dramatic changes to the epigenome. In endothelial cells (ECs), transient high-glucose levels resulted in sustained activation of the NF-kB subunit p65, which was associated with increased pro-inflammatory consequences, as measured by increased expression of pro-inflammatory molecules such as monocyte chemoattractant protein 1 (MCP1) and vascular cell adhesion molecule 1 (VCAM) [25]. The underlying mechanisms were traced to the H3K4 methyltransferase Set7, which controlled the sustained vascular gene expression patterns in response to transient hyperglycemia [26].

In vascular smooth muscle cells (VSMCs) derived from T2D-like *db/db* mice, increased inflammatory gene expression was observed, which was sustained in culture [27]. To discern underlying mechanisms, chromatin immunoprecipitation (ChIP) assays were performed and revealed that levels of H3K9me3 epigenetic modification were decreased at the promoters of inflammatory genes in *db/db* vs. control *db/+* VSMC in culture and that the cells from the *db/db* mice were more sensitive to upregulation of inflammatory genes in the presence of tumor necrosis factor alpha (TNFα). In parallel with these findings, it was reported that the levels of the H3K9me3 methyltransferase Suv39h1 were reduced in the *db/db* vs. the control cells; upon overexpression of Suv39h1, the inflammatory hyperstimulation state was reversed in the *db/db* VSMC, indicating that hyperglycemia and diabetes reversed a protective epigenetic histone modification signature in these cells [27].

These seminal observations in vascular cells have been complemented by studies in myeloid and whole blood cells. As these peripheral blood-derived cell types are generally more accessible in human subjects than vascular cells, stored cells from the DCCT-EDIC study have been employed for these studies in epigenetics and have shed key light on the molecular mechanisms of the long-lasting effects of high glucose.

### Myeloid and Whole Blood Cells

As the landmark DCCT/EDIC study for T1D provided important insight into the probability of a diabetes/high glucose-induced “metabolic memory,” peripheral blood cells available from the enrolled subjects during various periods of their journey through the years of study provided a critical framework for the identification of epigenetic mechanisms in these cells that could explain persistence of inflammation. In a recent study, available whole blood obtained at EDIC study baseline from 32 subjects (cases, conventional therapy, with complications) and 31 subjects (controls, intensive treatment, without complications) in overall study year #10 was subjected to studies of the epigenome. Furthermore, DNA-me was studied in blood monocytes of the same patients obtained at study years 16–17 [28]. From the whole blood, epigenome analyses revealed that 153 loci were found to display hypomethylation and 225 displayed hypermethylation (fold-change > 1.3 in case vs. controls), and in the monocytes, 155 hypomethylated and 247 hypermethylated loci were identified [28]. There were 12 annotated differentially methylated loci which were found to be common in both the whole blood and the monocytes; among these was thioredoxin interacting protein (TXNIP). TXNIP, linked to oxidative stress, has been associated with diabetic complications [29, 30] and with atherosclerosis; in animal model studies, deletion of TXNIP in atherosclerosis-prone mice was shown to reduce atherosclerosis [31–33].

In a later study using samples from the DCCT/EDIC, 499 randomly selected whole blood samples from the end of the DCCT close-out period were subjected to study. Associations between DNA-me and mean HbA1c during the DCCT at 186 cytosine-guanine dinucleotides (CpGs) were identified (false discovery rate [FDR], < 15%), and of these, 43 were positively associated, and 20 were negatively associated with an FDR < 5% [34••]. Many of these CpGs were located near genes with known relationships to complications, such as TXNIP, which was again identified for its association with diabetic complications in this later study. Mediation analyses revealed that several CpGs together explained 68–97% of the association of mean-DCCT HbA1c with the risk of complications during the EDIC period and that this occurred through modification of enhancer activity at myeloid and other cell types [34••].

Focusing on human macrophages, a distinct study reported that hyperglycemia increased the expression of S100A8 and S100A9 in macrophages and that expression of another S100/calgranulin, S100A12 (or EN-RAGE, a ligand of the receptor for advanced glycation end products [RAGE]) [35], was sustained in human macrophages after glucose levels were returned to euglycemia [36•]. Mechanistically, it was found that hyperglycemia increased the acetylation of histones which were bound to the S100A9 and S100A12 promoters in the “M1” or pro-inflammatory macrophages in processes involving SMYD3 and SET7/9 [36•].

Collectively, these studies in whole blood, human monocytes, and human macrophages revealed pro-inflammatory gene expression changes in key white blood cells that might contribute to increased CVD observed in the hyperglycemic milieu of diabetes.

### High Glucose, Human Monocytes, and Links to Interferon Networks

THP1 cells, a cell culture line of human monocytes/macrophages, were used to test the effects of high-glucose exposure. Through RNA-sequencing (RNA-seq), it was shown that high-glucose exposure upregulated genes linked to the interferon (IFN) pathway, including interferon-stimulated genes (ISGs) and interferon regulatory factors (IRFs). Using ChIP assays, it was shown that high-glucose conditions led to higher H3K9Ac levels around the promoters of the high glucose-stimulated genes [37]. Similar findings were observed when primary human monocytes were exposed to high glucose and indicated that in this metabolic condition, a shift to increased ISGs and IRF pathways through active epigenetic changes was prompted [37]. It was also reported in that work that the pattern of high glucose-induced gene changes in human monocytes was similar to that in which an IRF7 network was shown to be highly relevant to T1D pathogenesis [38].

Recently, direct associations between high glucose, monocytes, and SARS-CoV-2 infection were reported; single cell RNA-seq data from bronchoalveolar lavage (BAL) fluid of COVID-19 patients and controls identified that several genes associated with “interferon (IFN) α/β signaling pathway” were upregulated in mild and severe COVID-19 patients vs. controls in all 6 clusters of monocytes [39]. It was shown that SARS-CoV-2 infects peripheral blood monocytes and enhances the expression of angiotensin-converting enzyme-2 (ACE2), thereby increasing SARS-CoV-2 infection. SARS-CoV-2-infected monocytes expressed higher levels of IFNα, β, and λ, TNFα, IL1β, and IL6. In hyperglycemia, sustained aerobic glycolysis in monocytes, through HIF-1α, promoted viral replication, cytokine production, and mediated T cell dysfunction and lung epithelial cell death in SARS-CoV-2-infected lung. Although epigenetic modifications were not directly addressed in that study, which was focused on the role of glycolytic metabolism as a key driver of monocyte inflammation, it is possible that pre-existing hyperglycemia may exacerbate the overall response of diabetic monocytes to superimposed SARS-CoV-2 infection [39].

In the section to follow, updates on the relationships between IFN signaling and diabetic CVD are considered.

## Interferon Networks and Diabetic Cardiovascular Disease

There are three types of IFNs: type I IFNs include those such as IFN-α and IFN-β; type II IFN is represented by IFN-γ; and type III IFN is represented by IFN-λ [40, 41]. Type I and II IFNs, which have been studied in CVD [42], signal through the Janus kinase (Jak) and signal transducer and activator of transcription (STAT) pathway [43, 44]. Type I and II IFNs have been linked to the pathogenesis of atherosclerosis [45–48], where they play roles in distinct types of immune cells and during distinct phases of the early and progressive atherosclerotic process [42].

In murine atherosclerotic plaques, the most abundant of cell types is the macrophage; a recent meta-analysis of murine atherosclerosis data collated the results of multiple studies in which combinations of mass spectrometry and single cell RNA-seq highlighted that there were 4 subsets of macrophages clearly elucidated from these approaches [49]. These clusters were classified as TREM2-positive foamy macrophages, resident macrophages (these populated the healthy aorta), inflammatory macrophages, and IFN-inducible cell (IFNIC) macrophages [49]. The IFNIC macrophages reflect a type I IFN signature and predominantly express *Isg15*, *Irf7*, *Ifit1*, and *Ifit3* [49]. These macrophages also express CCL2, which is a key ligand for CCR2 [49]. These data supported roles for the IFN pathway in macrophages in atherosclerosis.

In the specific context of obesity and insulin resistance, which is a harbinger for T2D, a recent report used proteomic approaches to identify a pro-atherogenic “macrophage sterol-responsive network,” which predisposed macrophages to the accumulation of cholesterol [50]. IFN-γ was predicted to regulate this network; mice devoid of myeloid IFN-γ receptor (*Ifngr1*) (through bone marrow transplantation approaches) and the low-density lipoprotein receptor, *Ldlr*, displayed reduced macrophage cholesterol accumulation and atherogenesis. These effects were only noted in hypercholesterolemic *Ldlr* null mice with obesity and insulin resistance, but not in mice devoid of the *Ldlr* in the absence of obesity and insulin resistance [50].

Recently, in studies of the regression of diabetic atherosclerosis, which was probed in T1D-like streptozotocin-induced diabetic mice devoid of the *Ldlr*, novel roles for RAGE in regulation of IFN signaling in macrophages in these processes were uncovered [51••]. In that work, an aorta transplant model was used to test the roles of lesional vs. recipient macrophages in the regression of diabetic atherosclerosis, which has been shown to be impaired by diabetes [52–54]. Transplantation of aortic arches from diabetic, Western diet-fed *Ldlr* null mice into diabetic mice globally devoid of *Ager* (*Ager*, the gene encoding RAGE) vs. wild-type diabetic recipient mice resulted in significantly accelerated regression of atherosclerosis. In order to probe the underlying mechanisms, macrophages were sorted from the regressing plaques; in that study, CD45.1 macrophages were recipient-derived and CD45.2 macrophages were derived from the donor arches of the diabetic mice devoid of the *Ldlr*. RNA-seq experiments on the CD45.1 vs. CD45.2 macrophages linked RAGE-dependent mechanisms principally to the recipient macrophages and, for the first time, linked RAGE to interferon signaling in diabetes [51••]. Deletion of *Ager* in the regressing diabetic plaque environment downregulated macrophage expression of *Irf7*. Experiments in bone marrow-derived macrophages (BMDMs) were performed to directly test roles for RAGE ligands and RAGE in these processes and showed that RAGE ligand advanced glycation end products (AGE) upregulated expression of *Irf7*, which was prevented by either deletion of BMDM *Ager* or by siRNAs to reduce *Ager* expression in BMDMs. Critically, the studies uncovered key roles for IRF7 in regulation of inflammation and cholesterol metabolism, as knock-down of *Irf7* (using siRNAs) in BMDMs stimulated a switch from pro- to anti-inflammatory gene expression and regulated multiple genes linked to cholesterol efflux and homeostasis [51••]. In addition, the plasma of the recipient T1D-like wild-type and *Ager* null mice were assayed for the levels of IFN-γ; these were significantly lower in the diabetic *Ager* null vs. the wild-type recipient mice [51••]. Hence, the results of these studies spur new hypotheses and experiments to directly test the role of myeloid IRF7 in diabetic atherosclerosis, both in the progression and regression phases.

Finally, a recent study employed a therapeutic approach to block IFN signaling [55], through suppressor of cytokine signaling (SOCS) proteins, which have been shown to negatively regulate the Janus kinase (JAK)/STAT proteins that are downstream of IFN signaling [56, 57]. Streptozotocin-induced T1D-like state was induced in atherosclerosis-prone mice devoid of *Apoe*; these mice were treated with a SOCS-1 peptide or a control, mutant peptide for 6–10 weeks. Compared to mutant peptide, the mice treated with SOCS-1 peptide displayed reduced atherosclerotic lesion areas during both the early and advanced stages of lesion development; the plaques demonstrated significant reductions in the content of lipids, macrophages, and T cells but higher amounts of collagen and SMCs. In the systemic sites, evidence for reduced inflammation was observed by a reduction of the circulating Ly6C^HI^ monocytes and reduced expression of proinflammatory molecules [55]. Of note, there was no effect of the peptide on metabolic endpoints, suggesting that the benefits were likely mediated through interruption of JAK/STAT signaling.

Collectively, these updates in IFN pathobiology in insulin resistance and diabetes highlight new crossroads linking mechanisms of inflammation and disruption in cholesterol homeostasis to CVD. In the section to follow, this review examines recent updates in mitochondrial metabolism and showcases new insights by which diabetes impairs mitochondrial health and homeostatic properties and leads to CVD.

## Diabetes and Consequences for Mitochondrial Health — Pathways to Cardiovascular Diseases

Recent advances in understanding the mechanisms of and consequences associated with mitochondrial dysfunction in diabetes have used multiple approaches to shed new light on how diabetes adversely affects the heart and peripheral arterial system.

During diabetes, cardiac glucose uptake and oxidation are reduced, fatty acid oxidation is increased, and mitochondrial dysfunction is increased [58, 59]. However, the benefits or adverse consequences of such adaptations are not clear. Recent studies used transgenic mice that rendered T1D-like streptozotocin (or control) and that inducibly overexpress the glucose transporter GLUT4 in cardiomyocytes. First, in the non-diabetic mice, increased uptake of glucose decreased mitochondrial ATP generation, and by echocardiography, the mice displayed evidence of diastolic dysfunction [60]. In animals with pre-established T1D, inducible expression of GLUT4 in cardiomyocytes drove mitochondrial oxidative dysfunction. Transcriptomic analyses showed that the diabetes and elevated glucose uptake exerted their greatest effects on genes linked to mitochondrial function, and the underlying mechanisms of glucotoxicity were traced to *O*-GlcNAcylation of Sp1 transcription factor and direct *O*-GlcNAcylation of many electron transport chain subunits and other mitochondrial proteins, which collectively, caused mitochondrial dysfunction and were accompanied by echocardiographic evidence of myocardial dysfunction [60].

The consequences of hyperglycemia on inhibition of AMP-activated protein kinase A2 (AMPK-A2) on the induction of cardiomyopathy were recently traced to effects on mitochondria-associated endoplasmic reticulum membranes (MAMs), with particular focus on the roles of FUN14 domain containing 1 (Fundc1), which is an outer mitochondrial membrane protein and MAMs [61]. In the hearts of diabetic patients, levels of FUNDC1 were significantly higher than those of non-diabetic hearts, and in murine neonatal cardiomyocytes, high-glucose conditions also increased FUNDC1 and MAMs. In the cells, when either the expression of FUNDC1 or the inositol 1,4,5-trisphosphate type 2 receptor (Ip_3_r2) was reduced in high-glucose conditions, this caused inhibition of MAM formation, reduced endoplasmic reticulum-mitochondrial Ca^2+^ flux, and improvement in mitochondrial function. When FUNDC1 expression was ablated in cardiomyocytes in diabetic mice, this resulted in reduction in MAM formation, prevention of mitochondrial calcium increases, mitochondrial fragmentation, and apoptosis; in parallel, mitochondrial functional capacity and cardiac function were improved [61]. In mouse neonatal cardiomyocytes, high glucose suppressed AMPK activity and the overexpression of a constitutively active mutant of AMPK blocked high glucose-induced MAM formation and mitochondrial dysfunction [61]. These findings defined a new mechanism by which hyperglycemia caused mitochondrial and cardiomyocyte dysfunction through interruption of effect AMPK-A2 signaling.

Additional recent studies traced the adverse effects of high glucose on cardiomyocytes and in T1D-like streptozotocin mouse hearts to reductions in miR144; of note, plasma miR144 levels were found to be reduced in the plasma of diabetic patients who displayed cardiac dysfunction [62]. Through overexpression studies, the mechanisms of the protective effects of miR144 were shown to involve increased mitochondrial biogenesis and suppressed cell apoptosis at least in part through miR144-dependent effects on regulation of Rac1 [62].

In other studies, in mice with obesity or diabetes, levels of cardiac extracellular signal-regulated protein kinase 5 (ERK5) were found to be reduced; experiments to test the potential impact of this observation employed cardiomyocyte-specific deletion of *Erk5* in mice fed a high-fat diet; this resulted in reduction in cardiac contractility and increased mitochondrial damage with reductions in fuel oxidation and increased oxidative damage [63]. The mechanisms by which ERK5 regulated cardiac mitochondrial function were due, in part, to its effects on regulation of the Peroxisome proliferator-activated receptor-gamma coactivator-1alpha (PGC-1α); underlying mechanisms were traced to Gp91phox activation of calpain-1, which mediated degradation of ERK5 in free fatty acid-stressed cardiomyocytes. By contrast, the prevention of ERK5 loss through blockade of either Gp91phox or calpain-1 restored mitochondrial function [63].

Beyond the direct effects of high glucose and diabetes on cardiomyocytes and mitochondrial dysfunction, recent work has also addressed the effects of T2D-like diabetes on coronary EC mitochondrial calcium overload. In diabetes, one of the consequences of EC dysfunction is the loss of these cells due to apoptosis. Coronary EC apoptosis in diabetes results, in part, from the effects of mitochondrial calcium overload [64]. The potential role of hexokinase (HK) 2 was studied in these processes, on account of the role of this enzyme as an inhibitor of the voltage-dependent anion channel. Murine and human coronary ECs were isolated from T2D subjects; in the mice hearts, T2D was associated with reduced capillary density, and more apoptosis was observed in the ECs [64]. Levels of HK1 and HK2 were found to be significantly lower in the diabetic vs. nondiabetic coronary ECs, and the levels of mitochondrial calcium were higher in the diabetic vs. nondiabetic ECs as well. Similar findings regarding the levels of the HK forms were observed in the human diabetic coronary ECs when compared to the nondiabetic ECs. In the murine coronary ECs, treatment with high glucose but not high levels of fat reduced HK2 protein; when HK2 protein expression was restored in the diabetic murine coronary ECs, levels of HK2 arose, and mitochondrial calcium levels decreased. Importantly, the levels of mitochondrial reactive oxygen species (ROS) were reduced as well, altogether suggesting that HK2 reductions in diabetic coronary ECs drove pathological processes and mitochondrial dysfunction, which was linked to their demise [64].

In other studies focused on mitochondrial dysfunction and the diabetic endothelium, the role of mitochondrial fission in these cells was tested. Venous ECs isolated from patients with diabetes vs. healthy controls showed evidence of mitochondrial fragmentation in parallel with increased expression of Fission-1 (FIS1) protein [65]. To probe underlying mechanisms, human aortic ECs were subjected to high levels of glucose in culture (30 mM); this exposure condition resulted in increased expression of FIS1 and of dynamin-related protein 1 (DRP1); both proteins participate in mitochondrial fission. The altered mitochondrial dynamics in the presence of high levels of glucose were accompanied by increased mitochondrial ROS and impaired endothelial nitric oxide synthase responses [65]. Silencing of the expression of FIS1 or DRP1 reduced these adverse consequences in the high glucose-treated cells, which was not further improved by addition of an ROS scavenger, thereby suggesting that the processes of increased mitochondrial fission drove the impairment of endothelial function through increased mitochondrial ROS [65]. These findings on DRP1 were extended to a murine model of diabetic atherosclerosis (T1D-like streptozotocin-induced diabetes in *Apoe* null mice); in that work, treatment of these mice with a potent and selective inhibitor of DRP1, mitochondrial division inhibitor 1, reduced atherosclerosis in the diabetic mice. In parallel with reduced atherosclerosis, reductions in mitochondrial fragmentation, oxidative stress, endothelial dysfunction, and inflammation were observed, thereby providing evidence to support the premise that targeting mitochondrial fission and DRP1 in diabetes might be of benefit for atherosclerosis [66].

Recent insights into mitochondrial dysfunction in peripheral arterial disease are highly germane to diabetes, as peripheral arterial disease is an important cause of morbidity, amputations, and mortality in diabetes [67, 68]. For these studies, Polg^D257A^ mtDNA mutator mice were used; these are a model of multiple mitochondrial DNA mutations [69]. Strikingly, these mice were shown to be highly protected from hind limb ischemia (HLI) due to an adaptive enhancement of glycolytic metabolism and elevated ischemic muscle levels of 6-phosphofructo-2-kinase/fructose-2,6-biphosphatase 3 (PFKFB3). To further test this concept, BALB/c mice were used, as these mice are highly susceptible to the adverse effects of HLI when compared with C57BL/6 mice. To restore the function of PFKFB3 in the BALB/c mice, adenovirus-associated virus (AAV)-mediated overexpression in the limb muscles was used and shown to improve muscle contractile function and blood flow in the affected limb following HLI. RNA-seq data on muscle from patients with critical limb ischemia (CLI) revealed a unique deficit in the Reactome for “glucose metabolism.” The muscle tissue from patients with CLI displayed lower levels of the PFKFB3 protein, and in in vitro studies, the muscle progenitor cells showed decreased glycolytic flux capacity [70].

It is important to note that earlier studies demonstrated the direct key biochemical mechanisms by which hyperglycemia may exert its adverse effects, such as through glucose flux via the aldose reductase (AR) pathway, leading to opening of the mitochondrial permeability transition pore (MPTP) and modulation of the phosphorylation of glycogen synthase kinase (GSK)3-β pathways; both mechanisms lead to cardiomyocyte damage in diabetes [71, 72]. Additional mechanisms triggered by hyperglycemia include the formation of AGEs, which, via their chief signaling receptor RAGE, have been linked to nitrosylation of mitochondrial proteins and to apoptotic processes in the diabetic heart [73, 74].

Collectively, these examples of direct testing of mitochondrial abnormalities in the heart and peripheral vascular tissues in diabetes and ischemia highlight new mechanisms of CVD in diabetes and, potentially, novel therapeutic targets. In this context, recent enthusiasm has arisen around the broader benefits of sodium glucose co transporter 2 inhibitors (SLGT2 inhibitors) and GLP1R agonists for the treatment of diabetes. These agents imbue benefits to the cardiovascular system that appear to be beyond their ability to lower blood glucose levels. In the final section of this Review to follow, evidence regarding these newer agents from animal models to human subjects vis-à-vis cardiovascular protection will be considered.

## SGLT2 Inhibitors and GLP1 Receptor Agonism — Broad Benefits in CVD

A recent meta-analysis report of 8 trials reporting on 77,242 patients enrolled in GLP1R agonists and SGLT2 inhibitor trials in which the primary outcomes were the composite of myocardial infarction, stroke, and MACE and hospitalization for heart failure, and progression of kidney disease concluded that both therapeutic strategies were equally effective in reducing MACE in patients that had established CVD [75••]. No effects were noted, however, in patients that did not have a history of established CVD. Furthermore, the SGLT2 inhibitors displayed a more marked effect on the prevention of hospitalization for heart failure. In addition, the beneficial effects on the progression of diabetic kidney disease were superior in the setting of treatment with the SGLT2 inhibitors [75••].

At this time, the mechanisms of action by which these two different classes of anti-diabetes agents exert their beneficial effects on CVD remain under active investigation. Studies testing the SGLT2 inhibitors suggest that at least one key benefit of these agents, as illuminated in mouse models of atherosclerosis, for example, is the reduction in circulating and lesional immune and inflammatory signals and oxidative stress, perhaps, in part to increased AMPK signaling and/or reduction in activation of factors such as NF-kB, the NLRP3-caspase-1 inflammasome, and the AGE-RAGE pathways and/or direct effects on immune cell fate [76–79].

Irrespective of the precise mechanisms of action of these agents in CVD, these agents provide opportunity for new means to reduce morbidity and mortality in T2D.

## Conclusions

**Fig. 1:**
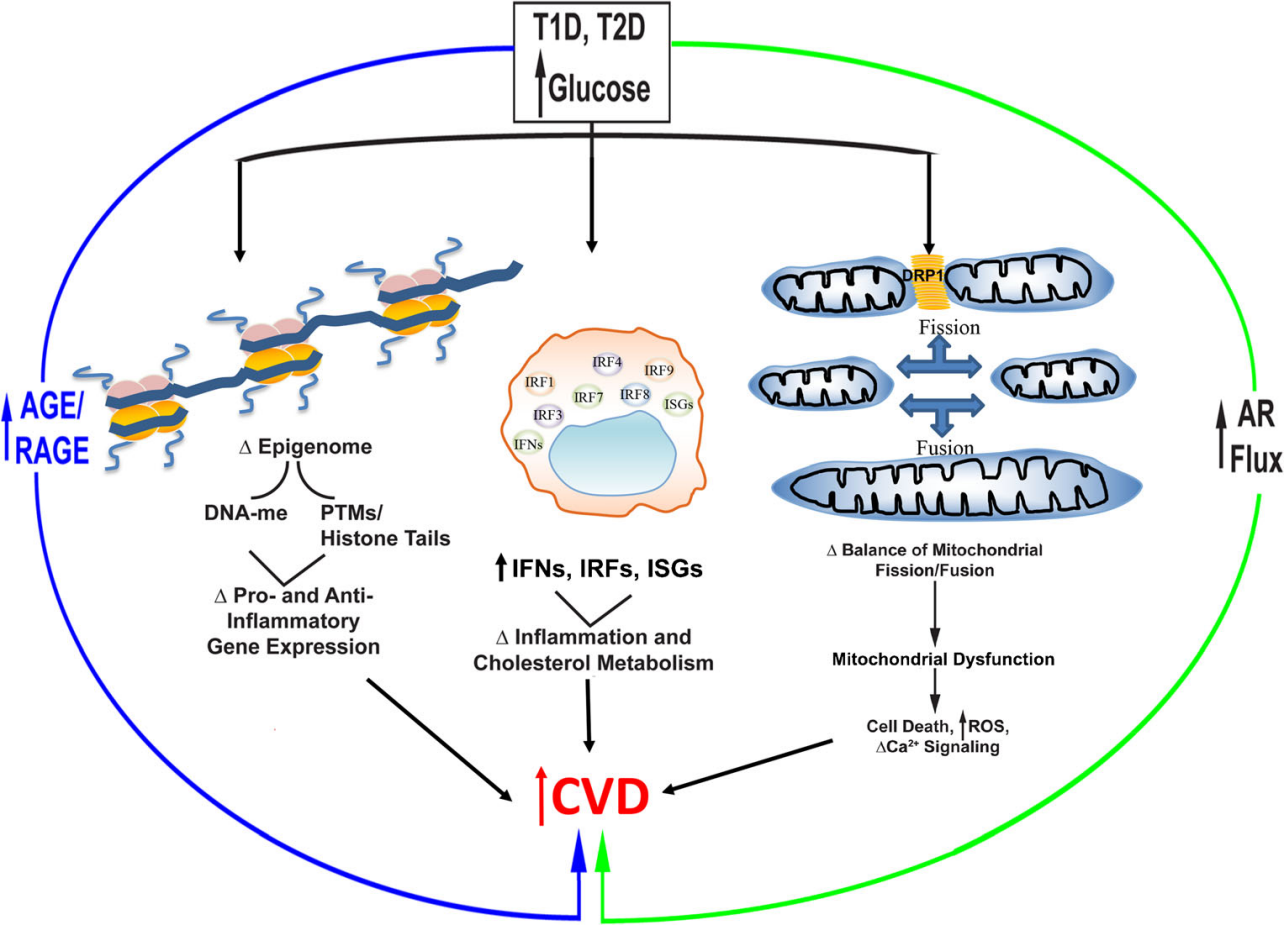
Updates in diabetes and CVD. This review focused on recent updates in mechanisms underlying accelerated CVD in diabetes. In the field of epigenetics, high levels of glucose exert a long-lasting metabolic memory on vascular and immune cells, leading to molecular signatures linked to diabetes and CVD. High levels of glucose and their consequent increased formation of AGEs, leading to activation of RAGE, upregulate multiple elements of the interferon pathways, such as IRFs, IFNs, and ISGs, and downstream pathways that lead to CVD. Finally, evidence continues to accrue linking high levels of glucose and pathways such as aldose reductase (AR) and AGE-RAGE to oxidative stress and mitochondrial dysfunction; such dysfunction affects a broad range of cell types that contribute to CVD. This review considered the evidence for cardioprotective roles for inhibitors of SGLT2 and for agonists of the GLP1 Rs in clinical studies. Additionally, recent evidence links downstream consequences of high levels of glucose, such as increased flux through the AR pathway and AGE generation/RAGE activation with CVD-provoking mechanisms. Collectively, these considerations highlight that research has led to new therapies and new targets for therapeutic intervention in diabetes to prevent/mitigate CVD. Abbreviations: AGE, advanced glycation end product; AR, aldose reductase; CVD, cardiovascular disease; DNA-me, DNA methylation; IFN, interferon; IRF, interferon response factor; ISG, interferon stimulated genes; PTM, post-translational modification; RAGE, receptor for advanced glycation end products; ROS, reactive oxygen species; T1D, type 1 diabetes; T2D, type 2 diabetes; Δ, differential; and DRP1, dynamin-related protein 1

This review focused on selected salient recent updates in research in diabetes and CVD (Fig. 1). In the field of epigenetics, recent work highlights direct mediating connections between DNA-me and diabetic complications, thereby opening up new avenues to interrogating mechanisms and therapeutic opportunities unveiled through the discovery of specifically affected genes, such as TXNIP. With respect to IFNs, research links the adverse effects of high glucose to regulation of IFN networks, including ISGs as well as some of the overarching IRFs. If and how such links between diabetes and hyperglycemia to IFNs may impact responses to viral infections, such as to SARS-CoV-2/COVID-19, remains to be studied. For certain, however, broad spectrum blockade of IFNs is not feasible given their roles in critical innate anti-viral defenses. Separating the beneficial from adverse IFN responses within the milieu of metabolic dysfunction will be a key challenge to overcome.

Further, recent spotlight on the mitochondria and their fission/fusion and interactions with endoplasmic reticulum, amidst the broad cast of molecules that regulate mitochondrial metabolism and oxidative stress, as examples, is highlighting new areas for therapeutic interventions in diabetes and CVD. Finally, recent clinical trials underscore that new classes of glucose-lowering agents, such as the GLP1 receptor agonists and related targets, and the SGLT2 inhibitors, impart significant cardioprotective (and renoprotective) effects in T2D. Yet, the promise of these agents is tempered by the reported potential adverse effects of these classes of agents, such as diabetic ketoacidosis. Their potential limitation of benefit only to T2D, at least at this time, also excludes the millions of persons suffering from T1D.

These recent research highlights open doors to new critical areas for study as strict control of hyperglycemia, and its anti-complications benefits are not perennial; once “metabolic amnesia” or the adverse effect of significant hypoglycemia ensues, physicians must be armed with distinct therapeutic approaches to counter the direct and indirect effects of hyperglycemia on CVD in diabetes. In conclusion, the recent research updates in epigenetics, IFN network signaling, and mitochondrial dysfunction in diabetes unveil the possibility of targeting these pathways, particularly in combination with glucose-lowering agents, for comprehensive treatment of diabetic CVD.
